# Quantitative photoconversion analysis of internal molecular dynamics in stress granules and other membraneless organelles in live cells

**DOI:** 10.1016/j.xpro.2020.100217

**Published:** 2020-12-10

**Authors:** Triana Amen, Daniel Kaganovich

**Affiliations:** 1 Department of Experimental Neurodegeneration, University Medical Center Goettingen, Goettingen 37073, Germany; 2 Base Pharmaceuticals, Boston, MA 02129, USA

**Keywords:** Cell Biology, Microscopy, CRISPR, Molecular/Chemical Probes

## Abstract

Photoconversion enables real-time labeling of protein sub-populations inside living cells, which can then be tracked with submicrometer resolution. Here, we detail the protocol of comparing protein dynamics inside membraneless organelles in live HEK293T cells using a CRISPR-Cas9 PABPC1-Dendra2 marker of stress granules. Measuring internal dynamics of membraneless organelles provides insight into their functional state, physical properties, and composition. Photoconversion has the advantage over other imaging techniques in that it is less phototoxic and allows for dual color tracking of proteins.

For complete details on the use and execution of this protocol, please refer to Amen and Kaganovich (2020).

## Highlights

• Photoconversion microscopy enables the measurement of protein dynamics in stress granules
• Protocol describes the construction of CRISPR-Cas9 stress granule photoconversion marker
• Protocol can measure the internal dynamics of other membraneless organelles in live cells

## Graphical Abstract

**Figure:**
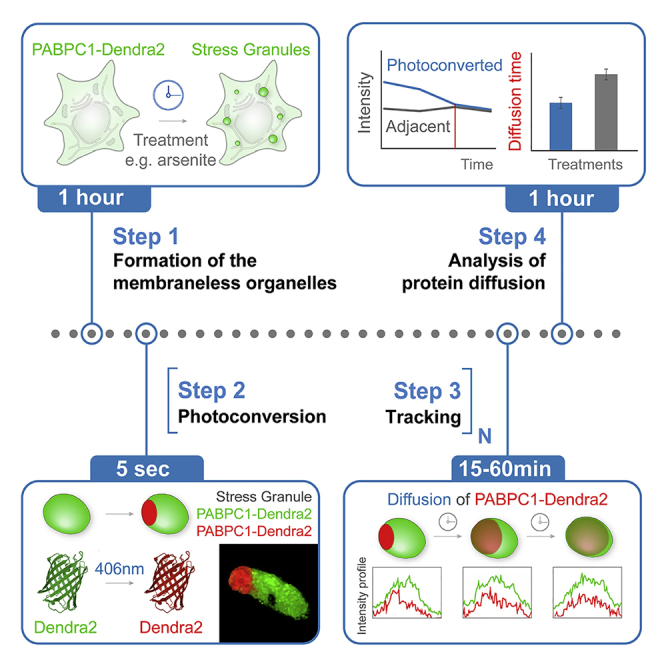

## Before you begin

### Creating a photoconvertible marker for a membraneless compartment

**Timing: 1 week to 2 months**

In this protocol we utilized a CRISPR-Cas9 tagged polyA-binding protein (PABPC1) fused to a photoconvertible fluorescent protein Dendra2 in HEK293T cell line to assess stress granule internal dynamics (Gurskaya et al., 2006, Ran et al., 2013, Chudakov et al., 2007a). PABPC1 is an abundant stress granule component (Aulas et al., 2017, Jain et al., 2016).

**Figure 1:**
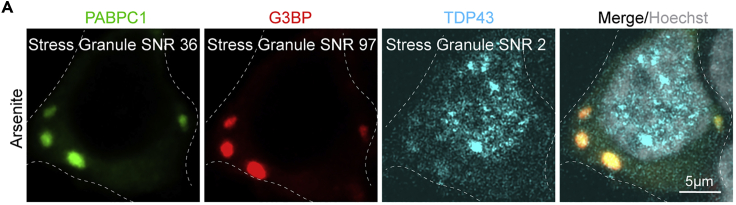
SNR ratio for stress granule components in HEK293T cells (A) Confocal microscopy of stress granules in HEK293T cells expressing CRISPR-Cas9 tagged PABPC1-Dendra, stained with anti-G3BP and anti-TDP43 antibodies, and treated with arsenite (200 μM, 2 h before fixation). SNR is shown for every component.

**CRITICAL:** It is necessary to use an easily detectable marker for the membraneless organelle. Low fluorescence intensity will lead to a low signal-to-noise ratio (SNR) and is difficult to detect. For example, PABPC1, G3BP, etc. will work best for the stress granule compartment in HEK293T cells whereas TDP43 will not provide sufficiently strong signal when using arsenite to form stress granules (Figure 1A).

***Note:*** We do not recommend overexpressing membraneless organelle markers when studying internal dynamics of its components because overexpression can dramatically change the kinetics of their formation and may also affect the composition and internal dynamics of the compartment (Kedersha et al., 2005, Kedersha et al., 1999). When overexpression is unavoidable, for example studying protein quality control compartments, or the effects of misfolded proteins (Kaganovich et al., 2008, Weisberg et al., 2012, England and Kaganovich, 2011), a stably integrated inducible marker with a strong uniform expression is recommended.

***Alternatives:*** If using a different marker is not an option and it does not provide sufficient SNR when tagged with a photoconvertible protein, one can fuse several photoconvertible tags to one marker in a row, creating a brighter construct. However, it needs to be noted that the size of the tag affects its diffusion (Gura Sadovsky et al., 2017), which is albeit less critical for a comparative analysis.

**Figure 2:**
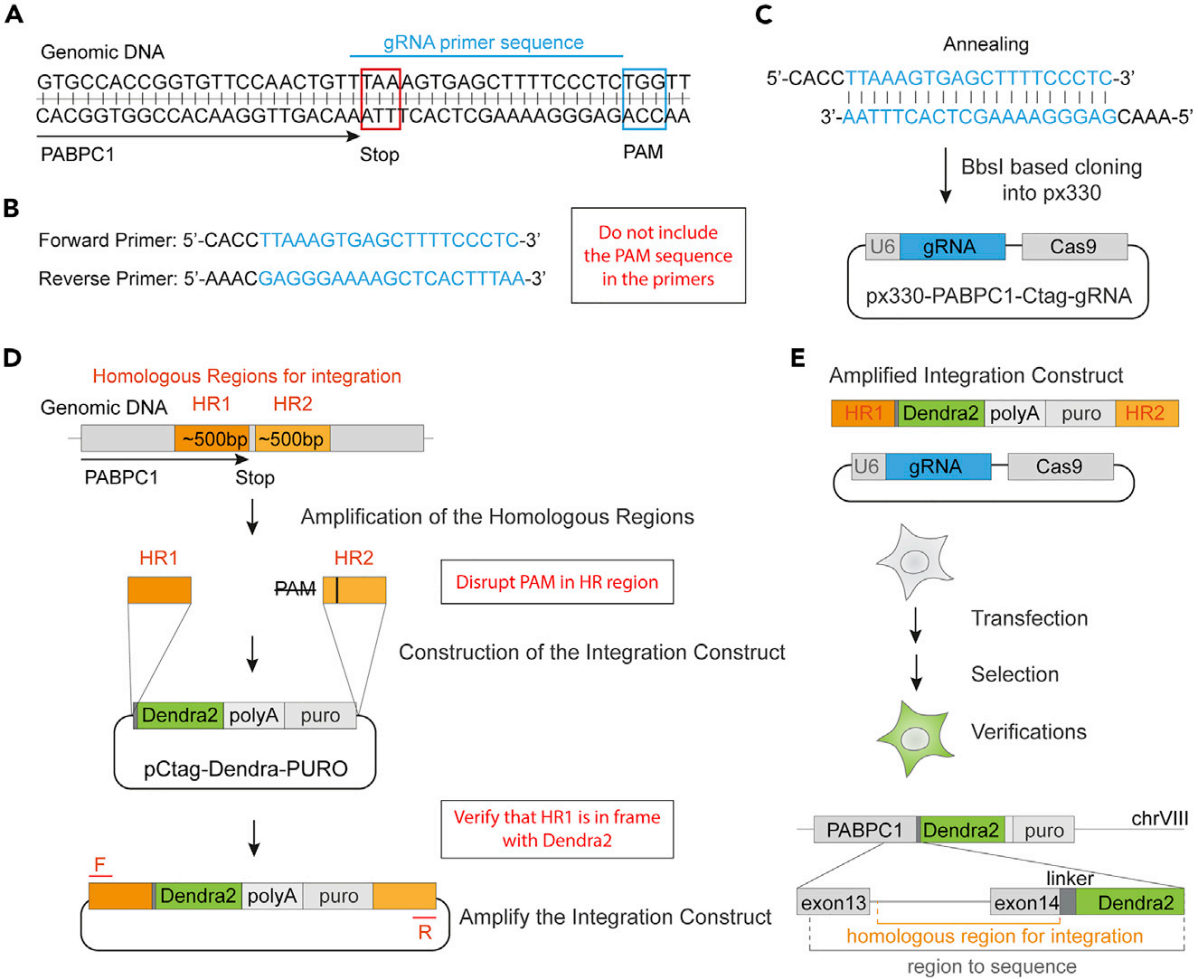
Construction of the PABPC1-Dendra2 cell line (A) Schematic of the target sequence selection and the gRNA primer design for the PABPC1 C-terminal tagging using px330 plasmid. (B) gRNA primer sequences. (C) Schematic of annealing and construction of the px330-gRNA plasmid for C-terminal tagging of PABPC1 in human cells. (D) Schematic of the integration construct construction. (E) Schematic of the PABPC1-Dendra2 cell line construction and verification of the genetic integration.
    ii. Write forward and reverse primers adjacent to but not including the PAM sequence (Figure 2B).
      **CRITICAL:** The “NGG” sequence of PAM is not included in the primers (Figure 2B).
    iii. Add sticky ends for the Bbs1 site of the px330 plasmid (5′ CACC- on the forward primer, and 5′-AAAC on the reverse primer) (Ran et al., 2013).
  b. Verify the specificity of primers using Digenome-Seq web tool (http://www.rgenome.net/cas-offinder/)(Bae et al., 2014) or other off-target finders. If off-targets are found, re-design primers to reduce off-targets: increasing the length of the primers reduces the chances of finding off-targets (we use 20–26 bp primers). Ideally you will find one match corresponding to your target.
  c. Design primers for the integration construct, which should contain regions homologous to the site of integration (HR), reading frame of the fluorescent protein, and can also contain a selection marker. We created a pCtag-Dendra2-PURO plasmid (Figure 2D, Supplementary Material 1) for C-terminal tagging and puromycin selection.
    i. Primers should amplify a ∼500 bp fragment of genomic DNA upstream and not including the stop codon sequence of PABPC1 (HR1, Figure 2D), and a ∼500 bp fragment downstream and not including the stop codon sequence.
    ii. Design restriction sites according to the site availability on the plasmid and HRs (Supplementary Material 1), we used EcoR1 and Kpn1 for HR1 (494 bp) and Sal1 and Pst1 for HR2 (522 bp).
    ii. Disrupt the PAM sequence by introducing a mutation in the primer sequence (Figure 2E), we changed the “TGG” PAM sequence to TCG when amplifying the HR2 (Figure 2D).
      **CRITICAL:** Make sure that the photoconvertible fluorescent protein reading frame is in frame with the gene of interest (HR1). You can insert a linker of your choice between the frames or use pCtag plasmid.
      **CRITICAL:** If the PAM sequence is inside of the coding region, destroy it by replacing one of the Gs by introducing a silent mutation.
  d. Design primers to amplify the integration construct, from the HR1 to the HR2 (F and R in Figure 2D); these are 15–20 bp blunt primers to amplify the construct for transfection.
  e. Design sequencing primers for the verification of the integration, we use primers upstream of HR1, and downstream of HR2 to verify the correct integration.

1. Design and order primers for the CRISPR-Cas9 or other genomic integration techniques.
  ***Note:*** The researcher should refer to the relevant genetic integration protocols, e.g., CRISPR-Cas9, suitable for their cell line and application (Ran et al., 2013, Sheff and Thorn, 2004, Amen and Kaganovich, 2017). Consult with an online guide, such as www.addgene.org/guides/crispr, to design the oligos. Use DNA databases, such as www.ensembl.org, to retrieve the genomic DNA sequences. For the endogenous tagging in mammalian cell line the user will need two plasmids: a plasmid with a specific guide RNA (gRNA) and Cas9 (Ran et al., 2013), and a construct carrying the photoconvertible fluorescent protein reading frame flanked by homologous regions for efficient genomic integration. Here we use an example of the construction of PABPC1-Dendra2 human cell line.
  a. Design two primers for the gRNA: forward and reverse, adjacent to but not including the protospacer adjacent motif (PAM) sequence.
    i. Choose the target sequence near the end of the PABPC1 genomic region on either side of the stop codon sequence (Figure 2A).

2. Construct plasmids for genetic engineering using standard protocols
  a. Anneal two primers from 1a and ligate the annealed oligos with a pre-cut with Bbs1 plasmid px330 (Ran et al., 2013) (Figure 2C). Proceed with bacterial transformation, amplification, purification, and sequencing of the plasmid. For sequencing use U6 promoter forward primer or a reverse primer downstream of the gRNA region.
    i. Primers from 1a (Figure 1C) are mixed at a 1:1 ratio, at a concentration of ∼10 μM. The mix is heated to 95°C for 5 min, followed by a slow decrease in temperature to 12°C over 30 min using PCR thermocycler.
      ***Alternatives:*** One can use a water bath to anneal the primers. Turn the water bath off after 5-min incubation at 95°C and wait for it to cool down.
  b. Construct the integration construct using standard restriction based cloning protocols or Gibson assembly protocols (Gibson et al., 2009) (Figure 2D)

3. Generate cell lines with a photoconvertible marker
  a. Transfect the 80% confluent cells with the Cas9-gRNA plasmid (2a) and a PCR fragment of the integration construct obtained with primers form 1d (Figure 2E).
    i. Amplify the integration construct using primers from 1d. Clean the PCR mix using the DNA cleanup kit.
    ii. Mix px330-gRNA plasmid and a cleaned PCR product (around 2 μg of each for a 10 cm plate transfection) in 500 μL of DMEM not supplemented with FBS, add transfection reagent per manufacturer instructions and let it sit for 10 min before mixing it in 10 mL of a ready media and adding it to 80% confluent HEK293T cells in a 10 cm plate.
      ***Note:*** The amount of the plasmid and a PCR fragment should be optimized according to the cell line and a transfection procedure used. We used 10 μL of RotiFect Plus (Carl Roth), 2 μg of the PCR product, and up to 2 μg of the px330-gRNA plasmid per 10 cm plate of HEK293T cells.
  b. Select the cells using an antibiotic selection or fluorescence-activated cell sorting (FACS). We introduced puromycin selection cassette on the integration construct, and thus selected clonal populations using loose splitting and recovering of resistant clones on the puromycin (2 μg/mL). This is a time-consuming process when working with mammalian cell culture, it takes 2–3 weeks to amplify the cells.
    i. Next day after transfection, change the medium to a puromycin containing medium.
      ***Note:*** The puromycin concentration needs to be optimized for the cell line.
    ii. In 1–2 days the cell death will be visible. Split the 1-cm plate loosely into 2–4 10 cm plates and wait for single cell clones to appear. The selection takes 2–3 weeks. Change the media to a fresh puromycin media at your discretion.
    iii. When single colonies contain 20–100 cells they can be picked to a 24-well plate to be amplified for verification and storage.
  c. Verify the integration in the clonal populations using western blot, immunofluorescence, sequencing (using primers constructed in 1e), and if possible functional assays.
    i. Use Western blot to screen the puromycin resistant clones, we used Dendra2 antibodies to screen for the integration and the size of the Dendra2 fusion.
    ii. Extract genomic DNA from the positive clones, amplify the regions of interest, upstream and including HR1 and downstream and including HR2, sequence cleaned up PCR products.
    iii. Score the stress granule formation.
    iv. Use immunofluorescence to monitor the stress granule markers.

***Note:*** There is a wide range of photoconvertible proteins, e.g., EosFP, Dendra2 (Wiedenmann et al., 2004, Gurskaya et al., 2006, Chudakov et al., 2007a). Photoconvertible fluorescent proteins should be chosen according to the user’s preference and microscope photoconversion capabilities. Consult with an online database, such as https://www.fpbase.org/, to compare the proteins (Lambert, 2019). Consider several parameters when choosing the photoconvertible proteins: some are more prone to bleaching (in our hands Dendra2 pre-photoconversion was more prone to bleaching than EosFP), some have a higher photoconversion rate, and dynamic range (on our system Dendra2 was superior to eosFP in its photoconversion rate), finally there are green to red (e.g., Dendra2, eosFP), or red to far-red photoconvertible proteins (Gurskaya et al., 2006, Chudakov et al., 2007a, Wiedenmann et al., 2004). We recommend testing the protein on your system before constructing the cell line, by overexpressing it on a plasmid.

***Note:*** Another factor that is important to consider when selecting the right fluorophore is the self-association tendency between fluorophore moieties. Dendra2 has a low self-association constant (Gurskaya et al., 2006); whereas fluorophores that do oligomerize (Costantini et al., 2012, Simeon et al., 2016) can artificially trigger the formation of membraneless organelles and other inclusions (Raghunathan et al., 2014).

***Note:*** If one wants to visualize a third marker when doing photoconversion, consider a far-red/near infra-red marker, as opposed to lower wavelength fluorescent proteins, such as TagBFP. Although imaging with a photoconversion laser (406 nm) using low laser power does not immediately photoconvert the proteins, it will interfere with the quantification, gradually adding to a photoconverted pool of proteins. Moreover, imaging with a blue laser causes photo-toxicity and is highly counterproductive to live-cell imaging.

### Cell culture preparation for live-cell imaging

**Timing: 24 h**

We followed standard cell culturing protocols for all experiments. HEK293T cells modified via CRISPR-Cas9 were maintained in the DMEM (Pan Biotech) supplemented with 10% fetal bovine serum (FBS), 1% penicillin/streptomycin, at 37°C/5% CO_2_. Puromycin (2 μg/mL, Sigma) selection can be omitted after a clonal endogenously tagged population was obtained and verified.

***Alternatives:*** Some imaging systems are sensitive to the media composition. If your microscope detects the signal from phenol red in the media, use phenol red free alternatives. Additionally, if your microscope is not equipped with a CO_2_ chamber, you can replace the media with a HEPES pH7.4 containing media.

4. One day before the experiment split the cells into a glass-bottom imaging plate. We use a 4-well 35 mm plates (Cellvis).

***Note:*** Splitting a day before the experiment allows the cells to recover. Adjust the timing of splitting according to your cell line. The goal here is to have a healthy cell line. Some cells, like stem cells, will require glass coating with an appropriate coating layer, e.g., Matrigel (Corning Life Sciences). You can choose to use a separate plate for each treatment, however having treatments in separate wells of the same plate consumes less time per repeat.

### Preparation of the imaging setup

**Timing: 1 h**

Imaging the dynamics of proteins inside cellular compartments requires high spatial resolution. Depending on the dynamics of the proteins a high temporal resolution may also be needed. For this protocol we used Nikon A1r confocal microscope equipped with a GaAsP photomultiplier modules, a CO_2_ chamber, a temperature control unit, and capable of photoactivation. The photoconversion was done in a bi-directional resonant scanning mode 512∗512 pixels, using a 406 nm laser (Coherent, 50 mW) pulses of 100 ms with a 10% of laser power.

***Note:*** It is recommended to make a test run of your photoconversion parameters before starting the experiments. You can change the duration of the pulse, or the power of the laser. You can use several pulses to photoconvert a portion of the membraneless compartment.

The tracking of the photoconverted proteins was done in a slower higher-resolution galvanometer scanning mode using CFI Plan Apo Lambda 60× oil objective, 488 nm laser (Coherent, 50 mW), and a 561 nm laser (Coherent, 50 mW).

**Figure 3:**
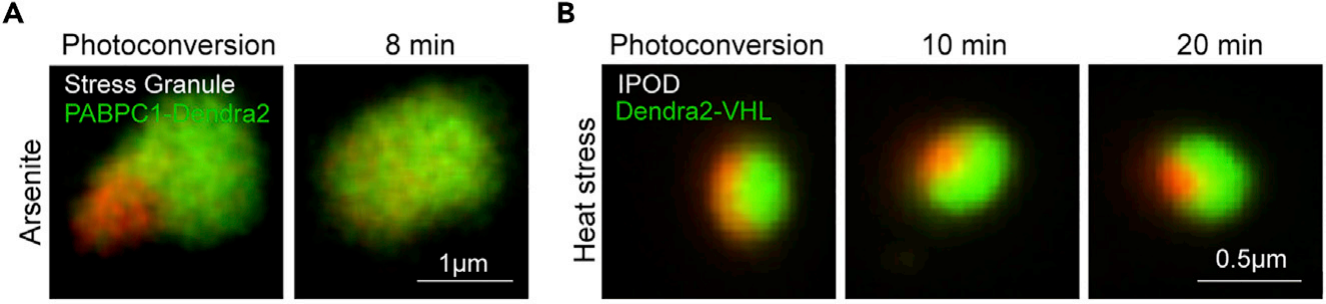
Diffusion of proteins inside membraneless compartments (A) Confocal microscopy of the diffusion of the photoconverted PABPC1-Dendra in HEK293T cells treated with arsenite (200 μM for 1 h). (B) Confocal microscopy of the photoconverted IPOD in *S. cerevisiae* yeast expressing Dendra2-VHL after 90 min of 38°C heat shock.

***Note:*** In this protocol we measured dynamics of the stress granule component, the dynamics can differ for another component used, between conditions, and between membraneless compartments (Figures 3A and 3B). Thus, it is important to test the approximate timing of the protein diffusion before the experiment. If you deal with the fast diffusion on a scale of seconds (what you would expect of the cytoplasmic protein), adjust your protocol using photoconverted intensity profile expansion (PIPE) method (Gura Sadovsky et al., 2017).

5. Initiate the system. Ensure that the CO_2_ and temperature are stabilized before introducing the cells to the microscope.

**CRITICAL:** It is important to verify that cells are healthy throughout the time of experiment by visual inspection and that the pH and the temperature of the media are maintained, using control units for parameters and visual inspection of the media coloring if using phenol red. For that we used temperature and CO_2_ controllers (OKO Lab).

## Key resources table

**Table:** 

REAGENT or RESOURCE	SOURCE	IDENTIFIER
**Chemicals, peptides, and recombinant proteins**		

Sodium arsenite	Sigma	7784-46-5

**Experimental models: cell lines**		

PABPC1-Dendra2-puromycin HEK293T	Kaganovich Lab	N/A

**Experimental models: organisms/strains**		

Yeast *Saccharomyces cerevisiae* BY4741 Dendra2-VHL	Kaganovich Lab	N/A

**Software and algorithms**		

Matplotlib	(Hunter, 2007)	https://matplotlib.org/gallery/lines_bars_and_markers/barchart.html
NIS Elements Software 4.10.04	Nikon	https://www.microscope.healthcare.nikon.com/products/software/nis-elements

**Other**		

Nikon A1r confocal microscope	Nikon	https://www.nikon.com/products/microscope-solutions/lineup/confocal/a1/
Cell culture incubator Forma Steri-Cycle	Thermo Fisher Scientific	https://www.thermofisher.com/order/catalog/product/381
4-chamber glass-bottom plates	Cellvis	D35C4200N
CO_2_ unit	OkoLab	DGTCO2BX
Temperature control unit	OkoLab	H201-T

## Materials and equipment

***Alternatives:*** This protocol uses Nikon A1r confocal microscope equipped with live-cell imaging chamber for the photoconversion and tracking of proteins inside living cells. Any other confocal microscope which is capable of photoactivation, and live-cell imaging can be used instead. We recommend using a microscope equipped with Electron Multiplying Charge-Coupled Devices (EMCCD) detectors to increase the SNR.

***Alternatives:*** We plot the analyzed data using python interface and matplotlib library (Hunter, 2007). Any other plotting tools, e.g., GraphPad Prism, can substitute.

***Alternatives:*** We used NIS Elements Software (Nikon) to calculate the fluorescence intensity and analyze the data. As an alternative image analysis software use ImageJ (Schneider et al., 2012), it has an ND2 plugin to import Nikon microscope generated images. (https://imagej.nih.gov/ij/plugins/nd2-reader.html).

The following solution is used to induce stress granules (time 1 h):

**Table:** 

Stress granule induction solution	Final concentration (μM)	Volume
Sodium arsenite (100 mM) in water	200 μM	1 μL/well
Cell culture media	n/a	0.5 mL
**Total**	**n/a**	**0.5 mL**

**CRITICAL:** Sodium arsenite is a highly soluble toxic component (Greenberg et al., 1979), refer to safety data sheet and take needed precautions when handling it.

## Step-by-step method details

### Formation of the membraneless compartment

**Timing: 1 h**

**Figure 4:**
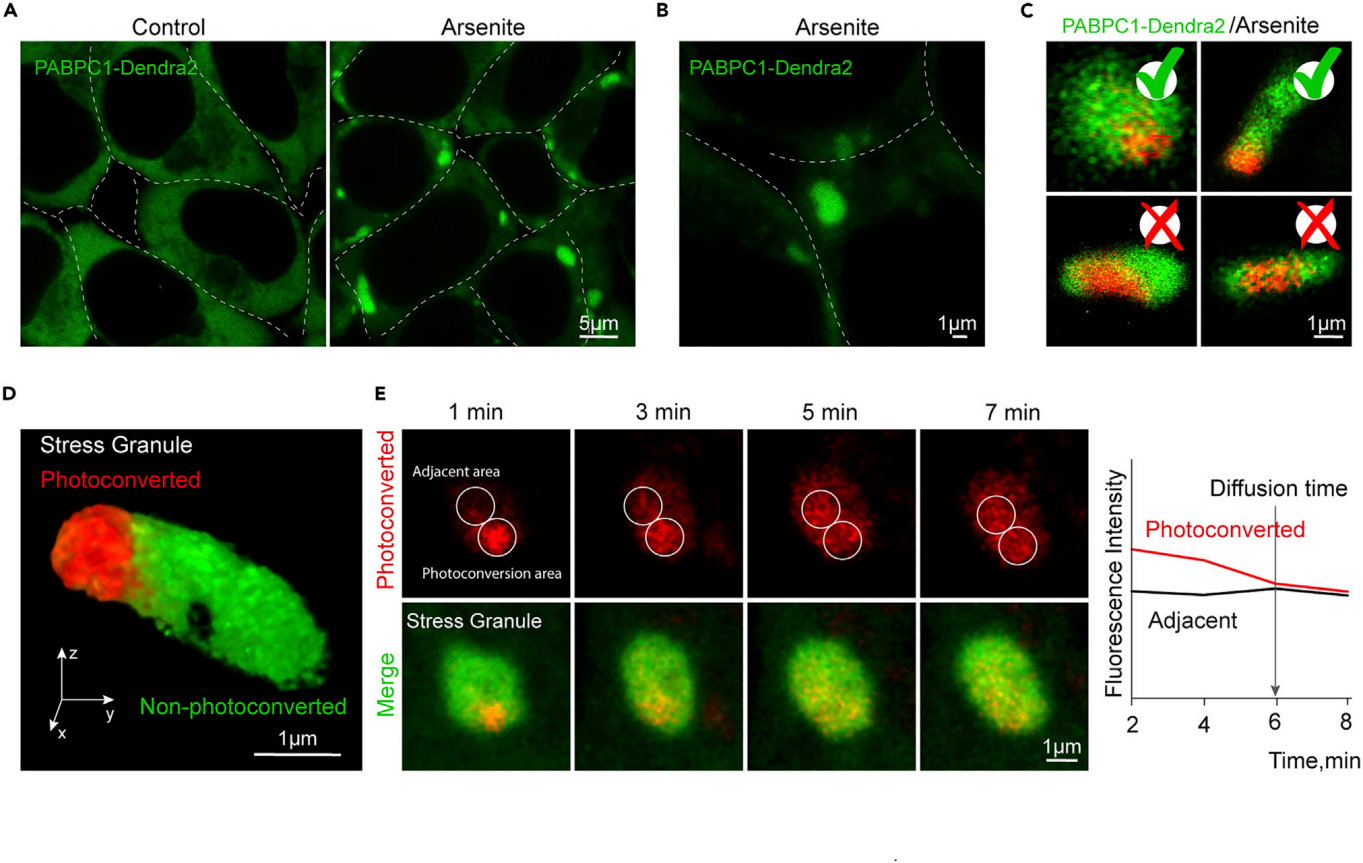
Protocol steps (A and B) Confocal microscopy of stress granules in HEK293T cells expressing PABPC1-Dendra2 during arsenite treatment (200 μM, 1 h). (C) Example of photoconverted stress granules. (D) 3D reconstruction of the photoconverted stress granule. (E.) Confocal microscopy of the tracking of the photoconverted pool of proteins inside the stress granule.

In this protocol we focus on the formation of stress granule compartments using sodium arsenite (Farny et al., 2009) (Figure 4A). Other membraneless compartments can require different conditions, e.g., heat stress for the protein quality control compartments (Figure 3B) (Hill et al., 2016, Spokoini et al., 2012). Yet others are constitutive, e.g., P bodies (Rao and Parker, 2017, Luo et al., 2018). Thus, you can substitute this step with the specific conditions.

***Note:*** This protocol can be used for a comparative analysis, thus the user can form the compartment in different conditions with the same treatment, or with different treatments.

1. Replace the media in the glass-bottom plate for live-cell imaging with the stress granule induction solution (200 μM arsenite in the media) and incubate for 1 h in the cell culture incubator at 37°C/5% CO_2_.

***Note:*** One can use the media in the wells and just mix it thoroughly with the arsenite and carefully pour it back in the well. Avoid starving the cells, as this will cause stress granule formation (Reineke et al., 2018), a freshly split cells should be used. Alternatively change the media to a fresh media before the experiment. We do not recommend introducing media perturbations before the experiment.

2. Introduce the plate into a live-cell imaging chamber of the microscope preheated to 37°C.

### Photoconvert a portion of the membraneless compartment

**Timing: 5 min initial setup, 5 s for the photoconversion**

Photoactivation, changing the excitation/emission spectra of a fluorescent protein using irradiation, is used in a variety of microscopic techniques. The photoswitching mechanisms often involve light induced trans/cis isomerization of the chromophore resulting in a red shift or activation of the spectra (Chudakov et al., 2007b, Habuchi et al., 2006). Reversible and irreversible photoactivation are techniques used in super resolution approaches, such as PALM (Betzig et al., 2006, Andresen et al., 2005, Cho et al., 2016), irreversible photoconversion of Dendra2 is used for photolabeling and tracking of proteins over extended periods of time (Chudakov et al., 2007a, Chudakov et al., 2007b). Some fluorescent proteins can be turned on from a non-fluorescent state, known as kindling, while others shift their excitation/emission spectra upon irradiation (Chudakov et al., 2003). Photoconversion of Dendra2 induces a red shift of fluorescence emission and excitation maxima after irradiation with a short pulse of a lower wavelength light, e.g., 406 nm. Photoconverted protein can thus be detected separately and simultaneously with a non-photoconverted pool of proteins (Chudakov et al., 2007a, Zhang et al., 2007). Photoswitching of Dendra2 is irreversible and thus can be used to track protein diffusion and protein degradation (Zhang et al., 2007, Gura Sadovsky et al., 2017, Chudakov et al., 2007a). The goal here is to irreversibly photoconvert a small portion (<half) of the membraneless compartment.

3. Prepare the setup for the photoconversion of stress granule compartment
  a. Zoom in on to the cell, that stress granule compartment is clearly visible and in the center of view. We use maximal zoom 8 (Figure 4B).
  b. Position the photoactivation point on the stress granule, if cell moves you can generally manually adjust it at the beginning of imaging.
    ***Note:*** Make sure the digital point is aligned with the actual photoconversion position. If not contact the technician to realign the system or adjust it manually using the software.
  c. Set the photoconversion parameters: laser power, the timing of the pulse, the number of pulses, the onset of the pulse. We set the following parameters: manual initiation of the photoconversion, pulse duration, 100 ms; number of pulses, manually curated.

***Note:*** The parameters should be optimized for your system and will depend on the SNR and the power of the laser.

4. Photoconvert the portion of a stress granule by starting the acquisition and initiating the photoconversion. Visualize both pre-photoconversion (488 nm excitation) and post-photoconversion (561 nm excitation) channels during the acquisition (Figure 4C, Methods Video S1).

***Note:*** Use lowest resolution to minimize the bleaching, the goal is to photoconvert a part of the compartment fast.

***Note:*** Even though we acquire a 2D acquisition during photoconversion and photoconvert a single point, we still photoconvert a volume of the stress granule, thus when tracking a small change in Z position will not affect the data (Figure 4D).

**CRITICAL:** It is important to use the same photoconversion parameters and timing of the acquisition between repeats and conditions. If it is not possible to control it manually, set a protocol in the system: photoconversion in 1 s after the start of the movie (allows you to record a pre-photoconverted state), pulse of 200 ms, duration of the acquisition 5 s.

Supplementary Material 1. pCtag-Dendra2-PURO plasmid sequence and map (open with SnapGene software)

### Track the diffusion

**Timing: 15 min to 1 h (the timing depends on the diffusion rate of your protein within the compartment, and the compartment state)**

In this step we will track the photoconverted proteins diffuse within the compartment over time. This step should immediately follow the photoconversion with a less than 5–10 s interval between the end of photoconversion and the beginning of tracking.

5. Set up a 2D time-lapse, acquiring an image every 1 minute for 15 min. Use the same position and magnification that was used for the photoconversion. Start the movie immediately after the end of the photoconversion (Troubleshooting 3).

***Note:*** We do not recommend continuous acquisition from the photoconversion due to the bleaching of the fluorescent protein. Photoconversion is done with a 30 frames per second rate, while the movie will only take 16 frames in 15 min, drastically reducing the bleaching. Also, it allows to utilize a slower higher-resolution imaging for the movie (Figures 4C and 4E).

***Note:*** Steps 1–6, from the formation/treatment of the membraneless compartment to photoconversion and tracking, are repeated N times for each treatment. To generate sufficient data for the calculations. After the first run and establishing the critical parameters for photoconversion and the duration of the tracking movie necessary to visualize the diffusion, the pipeline can be established, for example for a solidified stress granule compartment (which does not diffuse, manuscript in preparation) or the IPOD (yeast protein quality control compartment) (Kaganovich et al., 2008) we photoconvert 10 compartments, save the positions, and start a tracking movie for 10 points at the same time. For a faster diffusing stress granules (within 10 min) it is recommended to handle them one at a time repeating photoconversion-tracking step within a certain timeframe, for example 45 min after 1-h treatment for each condition.

**Pause Point:** The movies can be analyzed immediately or at a later stage when sufficient data points are collected from all the treatments.

### Quantify the diffusion time

**Timing: 5 min for one movie**

In this step we will use the tracking movies that were acquired in the previous step to quantify the diffusion of the protein across the stress granule compartment. For that we will use the NIS Elements Software (Nikon) to calculate the fluorescence intensity of the area of photoconversion and an equivalent adjacent area over time.

6. Quantify the fluorescence intensity of the photoconverted area and the immediately adjacent area along the long axis of the stress granule over the time of the movie (Troubleshooting 2), from 0 to 15 min (Figure 4E).

***Note:*** Diffusion time of the protein of interest across the membraneless compartment is the time it takes equalize the fluorescence intensity (5% margin) between the photoconverted area and the adjacent area of the same volume. The diffusion time often corresponds to a complete diffusion across the membraneless compartments (equalizing of the fluorescence of the photoconverted protein pool), however there are cases of elongated compartments where it does not.

7. Determine the diffusion time of the protein by plotting the line graphs of photoconverted and adjacent areas over time (Figure 4E).

***Note:*** we use a rather poor time resolution (±1 min), which is justified by two considerations. First, we reduce the bleaching, acquiring only 16 frames in 15 min. Second, there are cases of solidifications that increase the time of diffusion to 1 h, thus we do not need a resolution on a second’s scale. If, however, you are dealing with a fast-diffusing compartment (under a minute), or desire a higher resolved comparison, adjust the time-lapse parameters in step 5. For a higher resolved movie consider correcting for bleaching.

***Note:*** Using our parameters, the bleaching correction, although improves the data visuals, does not change the diffusion time. However, if increasing the time resolution, or dealing with a longer time-lapse (more frames) we recommend running the bleaching time-lapse with a fully photoconverted membraneless compartment (in addition to step 5) and correcting the data.

## Expected outcomes

Expected outcome is a table containing diffusion times (N repeats) for each treatment or condition that the user wishes to compare, that can be visualized with a simple bar graph or a box-and-whisker plot.

## Quantification and statistical analysis

Quantification of the diffusion time can be done by plotting the fluorescence intensity of the photoconverted and the adjacent areas on the graph (Figure 4E). The diffusion time is the first instance when the mean intensity of the photoconverted area equalizes with the adjacent area with a 5% margin. After the diffusion time is determined for each tracking movie (steps 6 and 7), the data are prepared for the statistical analysis. Exclusion of the data points can happen due to the loss of focus in the tracking movie, or drastic shape changes of the membraneless organelle (Troubleshooting 2 and 3). Normal distribution of the data can be verified using Shapiro-Wilk test and the equality of variances can be verified by Levene’s test. After that, P values are calculated by two-tailed Student t-test (2 conditions), or one-way ANOVA (>2 conditions).

## Limitations

This protocol describes comparative analysis of internal dynamics of stress granules. We describe the parameters used to compare the dynamics of PABPC1-Dendra2 protein fusion. The dynamics of other stress granule components may differ since membraneless compartments are not uniform in structure and composition (Aulas et al., 2017, Cirillo et al., 2020, Jain et al., 2016), and the parameters should be adjusted accordingly. This protocol is useful for abundant markers of membraneless organelles and will not be useful for a scarce component. One of the limitations is a fusion with a specific photoconvertible fluorescent protein that must be endogenously tagged, construction of the cell line may discourage the users. And finally, tagging of the components may interfere with the dynamics of the compartment, thus this protocol should be preferably used for a comparative analysis between the states of the compartment.

## Troubleshooting

### Problem 1

Insufficient or no signal when photoconverting.

There are three possibilities to consider: (1) the marker for the membraneless compartment is not abundant enough; an indication would be a low signal before photoconversion, (2) the photoconverting laser pulse is too weak or the user is using the wrong wavelength (3) the channel detecting the photoconverted signal is not optimized.

### Potential solution

(1) the user can try a different marker or improving the signal on the imaging system by installing a more sensitive detector.
(2) optimize the laser power by testing a range from the highest to the lowest.
(3) we recommend using a higher laser power when exciting the photoconverted signal, for example if you use 2% when detecting the signal before photoconversion (488 nm for Dendra2) use 4% for a post-photoconversion signal (561 nm, for Dendra2).

### Problem 2

Stress granule shapeshifts during photoconversion or time-lapse. Stress granules are not static, they change shapes, can divide, and fuse. It is normal to witness such an event in approximately 1% of cases during the tracking or photoconversion. However, it prevents the quantification.

### Potential solution

Usually it will not be possible to quantify, thus this will not contribute to the data.

### Problem 3

Stress granule moves out of focus during tracking. We utilize 2D time-lapse to track the photoconversion to minimize the bleaching.

### Potential solution

It is possible to do a 3D time-lapse, however we do not recommend it since it will increase bleaching and time of illumination. As a solution we recommend manually adjusting the Z position or discarding the data point as an outlier.

### Problem 4

Too much bleaching during the time-lapse.

### Potential solution

(1) Lower the laser power used for acquisition of movies during photoconversion and tracking
(2) Consider lowering time resolution, for example take a frame every 2 min instead of 1, the goal is to reduce illumination
(3) Optimize the speed of the scanning, faster speed with lower resolution will reduce bleaching and generally is recommended. The priority is to have good data and then pretty images.

### Problem 5

No diffusion detected.

### Potential solution

Consider optimizing the time scale. Some condition may affect the diffusion 10 times, to test that set a time-lapse with a step of 5 min for 2 h. Adjust the tracking time-lapse time accordingly. Consider photoconverting several compartments and setting up a multi-point time-lapse.

### Problem 6

Diffusion is immediate.

### Potential solution

Most likely you are dealing with a liquid-like compartment, such as cytoplasmic space. Use the extended photoconversion acquisition to quantify, refer to the PIPE method for the analysis (Gura Sadovsky et al., 2017)

### Problem 7

Proteins diffuse but fluorescence intensity of the photoconverted area is not equilibrating with the adjacent area.

### Potential solution

It is possible that stress granule fluorescence intensity is not uniform overall, compare the fluorescence intensity of the non-photoconverted signal (ex. 488 nm). We recommend quantifying the signal from the uniform stress granules, since a reduction of the signal during tracking can indicate the change of stress granule volume (refer to the Problem 2).

## Resource availability

### Lead contact

Further information and requests for resources and reagents should be directed to and will be fulfilled by the Lead Contact, Daniel Kaganovich (danielkaganovich@gmail.com).

### Materials availability

Cell line and the plasmids used in this study are available upon request.

### Data and code availability

The protocol does not generate code. We included all the datasets.

## Supplemental Information

Methods Video S1. Photoconversion of the stress granule compartment in HEK293T cells expressing CRISPR-Cas9 tagged PABPC1-Dendra2, related to step 4
