# Supplementary figures and images for: Quantitative photoconversion analysis of internal molecular dynamics in stress granules and other membraneless organelles in live cells
Source: STAR Protoc. 2020 Dec 10;1(3):100217. doi: 10.1016/j.xpro.2020.100217 (PMC7757677; doi:10.1016/j.xpro.2020.100217)

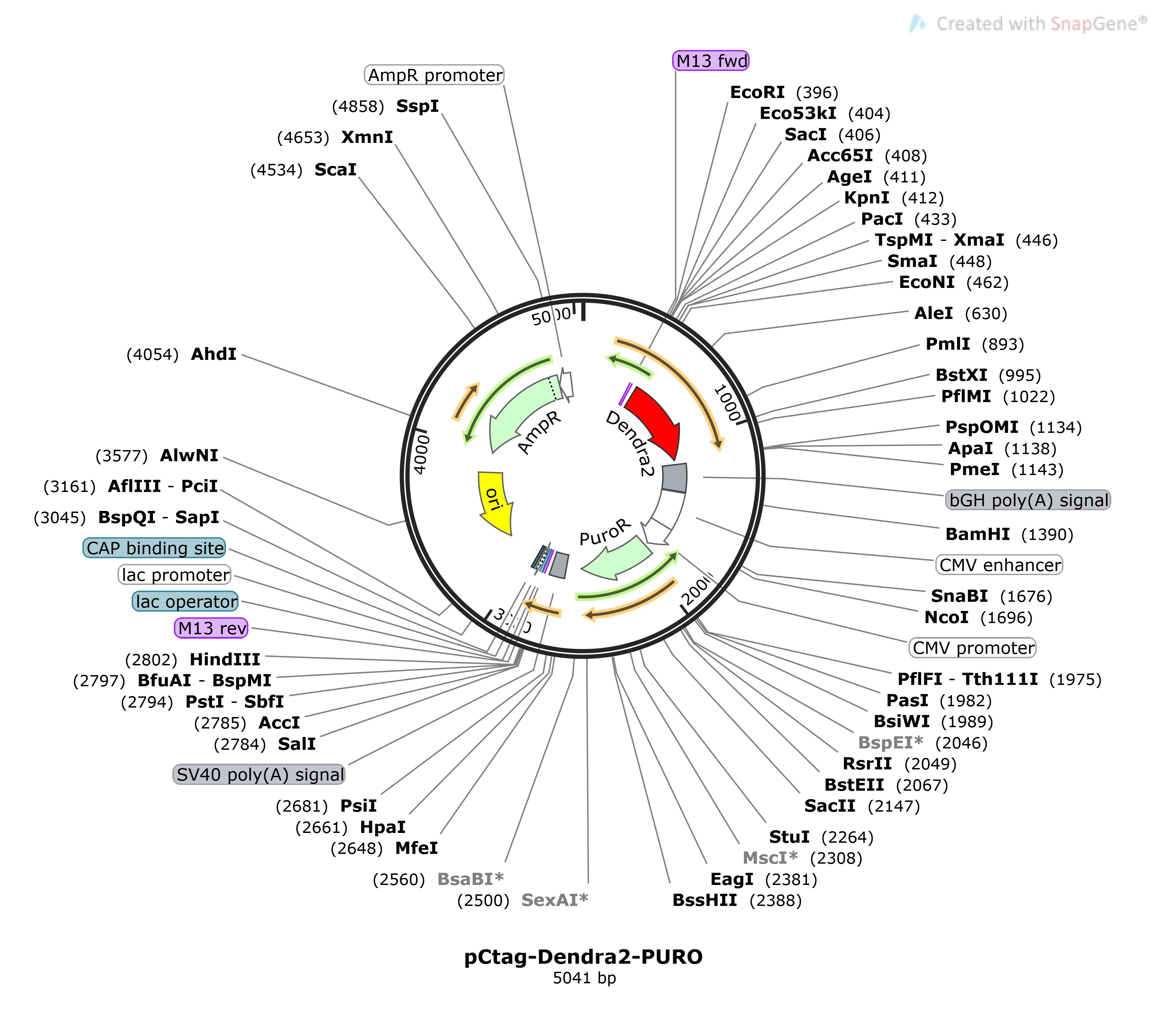

Supplement: Supplementary Material 1. pCtag-Dendra2-PURO plasmid sequence and map (open with SnapGene software) [file mmc1.jpg]
